# Integrated clinical and PBMC transcriptomic profiling identifies lipid metabolism-related candidate signatures associated with chronic brucellosis

**DOI:** 10.3389/fcimb.2026.1725128

**Published:** 2026-07-02

**Authors:** Rong Wang, Bin Niu, Xin Zhang, Yinghan Wang, Haiyan Tian, Chenming Zhang, Liaoyun Zhang

**Affiliations:** 1 Department of Infectious Diseases, The First Hospital of Shanxi Medical University, Taiyuan, Shanxi, China; 2 Graduate School, Shanxi Medical University, Taiyuan, Shanxi, China

**Keywords:** brucellosis, lipid metabolism, machine learning, peripheral blood mononuclear cells, PPAR signaling pathway, transcriptome analysis

## Abstract

**Objectives:**

To investigate clinical and peripheral blood mononuclear cells (PBMCs) transcriptomic features associated with chronic brucellosis and to test the hypothesis that lipid metabolism-related transcriptomic alterations in PBMCs may be involved in chronic disease progression.

**Methods:**

We analyzed 463 brucellosis patients (350 acute, 113 chronic) to compare clinical and laboratory features and to construct a multivariable logistic regression model for chronicity risk. RNA sequencing of PBMCs from 15 acute and 15 chronic cases identified differentially expressed genes, which were subjected to Gene Ontology (GO) and Kyoto Encyclopedia of Genes and Genomes (KEGG) enrichment analyses. Machine learning methods (LASSO regression and random forest) were used to select feature genes and build a diagnostic model. Immune cell infiltration and drug–gene interactions were further explored.

**Results:**

Chronic cases showed higher high-density lipoprotein cholesterol (HDL-C) and lower triglycerides, whereas acute cases had higher D-dimer, procalcitonin, and erythrocyte sedimentation rate (ESR). The clinical model incorporating 11 variables achieved an area under the curve (AUC) of 0.825. In an exploratory RNA-seq cohort comprising 30 PBMC samples from 15 acute and 15 chronic brucellosis patients, 2,775 exploratory candidate differentially expressed genes were identified, and enrichment analysis highlighted pathways related to fatty acid metabolism and peroxisome proliferator-activated receptor (PPAR) signaling. Three lipid metabolism-related feature genes, BDH1, CERS6, and DPEP3, showed high discriminatory performance in internal analysis, with an apparent AUC of 0.964. However, given the limited RNA-seq sample size and lack of external validation, this result should be interpreted cautiously. These genes were also associated with distinct immune infiltration patterns. Drug-Gene Interaction database (DGIdb)-based exploratory analysis suggested hypothesis-generating associations between CERS6 and several approved immunomodulatory agents, including TNF-α inhibitors.

**Conclusions:**

Chronic brucellosis may be associated with lipid metabolism-related transcriptomic alterations and immune-cell composition changes. The identified clinical and molecular features may provide candidate signatures for chronicity risk assessment and hypothesis generation, but require further validation in independent cohorts.

## 1 Introduction

Brucellosis, also known as Malta fever, is a significant zoonotic disease that poses a serious threat to public health and safety. The causative agents are bacteria of the genus *Brucella*, which was named in honor of David Bruce (1855–1931) (Gorvel, 2008). Patients in the acute phase of brucellosis commonly present with fever, night sweats, splenomegaly, peripheral lymphadenopathy, and orchitis. In contrast, the chronic phase is characterized predominantly by musculoskeletal pain, especially in the lower back or joints such as the spine and sacroiliac joints (Luo et al., 2025). Owing to its diverse and nonspecific clinical manifestations, together with considerable interindividual variability, brucellosis is prone to misdiagnosis or missed diagnosis, which may delay timely treatment and contribute to progression into chronic disease. Recent studies have further demonstrated that *Brucella* can persist within host cells and establish chronic infection (Pellegrini et al., 2025).

PBMCs are central mediators of systemic immune responses. They are widely employed in studies of autoimmune diseases (e.g., systemic sclerosis), chronic inflammatory disorders (e.g., COPD), tumor immune surveillance, as well as infection and vaccine responses. Applications span multiple levels, from cellular function and metabolic profiling to multi-omics analyses, and provide insights into immune microenvironment alterations and potential therapeutic targets under chronic pathological conditions (Sen et al., 2018; Agarwal et al., 2019; Szabo et al., 2023; Lehle et al., 2025). Yang et al. detected potential *Brucella* infection in PBMCs using immunofluorescence labeling, thereby demonstrating the presence of the pathogen within host cells (Yang et al., 2019).

Accumulating evidence indicates that metabolic reprogramming plays an important role in infectious diseases and can shape host defense, inflammatory responses, and disease outcomes. Recent studies have further highlighted that infection and sepsis can induce systemic physiological disruption and metabolic remodeling, underscoring the importance of host metabolism in infection pathogenesis (Willmann and Moita, 2024). Lipid metabolism is one of the key components of this process, providing structural support, energy provision, and immune regulation. Pathogens can manipulate host lipid metabolism to facilitate invasion, replication, and immune evasion, thus influencing the onset and persistence of infection (Prado et al., 2023). In tuberculosis models, *Mycobacterium tuberculosis* infection drives macrophages to accumulate cholesterol and form lipid droplets, which in turn support bacterial persistence (Long et al., 2022). In the context of viral infections, SARS-CoV-2–induced reprogramming of lipid and glucose metabolism has been implicated in the development of long-term symptoms and identified as a potential therapeutic target (Wang et al., 2024a). Advancing research in this field offers critical opportunities to elucidate the mechanisms of infectious pathogenesis and to identify novel therapeutic targets. Although lipid metabolic remodeling has been implicated in several infectious and inflammatory diseases, PBMC-specific lipid metabolism-related transcriptomic signatures associated with chronic brucellosis remain poorly characterized. Therefore, identifying candidate lipid metabolism-related transcriptomic features in PBMCs may provide new insights into the host response patterns associated with chronic brucellosis.

In this study, we integrated RNA sequencing with machine learning approaches to identify candidate biomarkers associated with chronic brucellosis and to explore their potential immunological relevance. RNA sequencing enables comprehensive analysis of transcriptomic alterations, allowing systematic identification of differentially expressed genes in PBMCs from patients with brucellosis. However, transcriptomic datasets typically contain thousands of gene variables but relatively limited sample sizes, creating challenges related to high dimensionality, feature redundancy, and potential overfitting. Therefore, robust feature-selection strategies are required to identify biologically meaningful and statistically stable candidate biomarkers. Feature selection has been widely applied in bioinformatics to reduce dimensionality and improve the interpretability of high-dimensional biological data (Saeys et al., 2007). To further optimize biomarker selection, we applied two machine learning algorithms: least absolute shrinkage and selection operator (LASSO) regression and random forest. LASSO regression was used to shrink less informative variables and select genes with non-zero coefficients (Friedman et al., 2010), while random forest analysis was applied to rank variable importance and further prioritize candidate feature genes. Random forest has been widely used for feature selection and biomarker discovery in high-dimensional genomic data (Chen and Ishwaran, 2012). Both methods have demonstrated strong robustness in handling high-dimensional transcriptomic data, while effectively reducing the impact of noise and outliers. Unlike many previous studies that relied primarily on public datasets, our analysis was conducted entirely on clinically collected PBMC samples, thereby enhancing its clinical relevance. Through this integrative strategy, we not only identified candidate genes with diagnostic potential but also uncovered their possible roles in the immune response to brucellosis.

## 2 Methods

### 2.1 Study design and participant selection

Between May 2019 and December 2023, 463 patients diagnosed with brucellosis were enrolled, comprising 350 acute and 113 chronic cases. The diagnosis of brucellosis was based on epidemiological history, clinical manifestations, and laboratory confirmation (serological agglutination test and/or culture). The diagnosis followed the criteria of the national guideline “Diagnosis for Brucellosis (WS 269-2019)” issued by the National Health Commission in 2019 (Liu et al., 2022; Wang et al., 2024a):

1. Epidemiological exposure, such as close contact with livestock or animal products suspected of carrying Brucella, or ingestion of unpasteurized dairy or undercooked meat.
2. Clinical symptoms including prolonged fever (low- or high-grade), excessive sweating, fatigue, arthralgia, or myalgia, some patients had lymphadenopathy, hepatosplenomegaly, or testicular swelling, while a few exhibited various rashes or jaundice.
3. Laboratory findings, including positive results of the rose bengal plate agglutination test (RBT), colloidal gold immunochromatographic assay (GICA), and enzyme-linked immunosorbent assay (ELISA). In addition, Brucella organisms were observed by Gram staining of cultured isolates.

A clinical diagnosis required meeting criteria 1) and 2), together with any one of 3) simultaneously. Patients were classified as chronic if symptoms persisted for >6 months (Jiang et al., 2019).

The study protocol was approved by the Ethics Committee of the First Hospital of Shanxi Medical University (NO. KYYJ-2025-143). Written informed consent was obtained or waived in accordance with national regulations. This study followed the STROBE and TRIPOD guidelines.

### 2.2 Sample collection and RNA extraction

For the transcriptomic study, PBMCs were collected from 30 patients, including 15 acute and 15 chronic brucellosis cases, prior to antimicrobial treatment. PBMCs were isolated using Ficoll-Paque density-gradient centrifugation. Total RNA was extracted with TRIzol reagent (Invitrogen, USA) following the manufacturer’s instructions. RNA purity and concentration were measured with a Nanodrop 2000 spectrophotometer (Thermo Scientific), and integrity was assessed using an Agilent 2100 Bioanalyzer. Only high-quality RNA samples were used for library preparation.

### 2.3 RNA sequencing

For each sample, 1 μg of RNA was used for library construction. mRNA was enriched with poly-T oligo-attached magnetic beads, fragmented, and reverse transcribed into double-stranded cDNA. After end repair, adaptor ligation, and PCR amplification, libraries were purified and qualified using an Agilent 2100 Bioanalyzer, and sequenced on the DNBSEQ platform (BGI-Shenzhen, China) to generate 150 bp paired-end reads. Raw reads were filtered to remove adapters, low-quality reads, and reads with excessive poly-N, yielding clean reads. Clean reads were aligned to the human reference genome (GRCh38) using HISAT2 (v2.2.1), and gene-level read counts were obtained with FeatureCounts (v2.0.1). RNA concentration was measured using a microplate reader, and RNA integrity was assessed using the Agilent 4150 system. Only RNA samples that met the library-construction and sequencing quality requirements were used for subsequent library preparation. Sequencing quality control was performed for all RNA-seq samples. The number of clean reads ranged from 34.74 to 91.38 million reads per sample, corresponding to 5.08–13.52 Gb of clean bases per sample. The Q30 values ranged from 93.54% to 98.74%, and the GC content ranged from 47.84% to 51.07%. The duplication rates ranged from 0.50% to 2.44%. Clean reads were aligned to the human reference genome, with total mapping rates ranging from 96.79% to 98.26% and uniquely mapped reads ranging from 88.15% to 94.95%.

### 2.4 Differential expression and enrichment analysis

Differential expression analysis between acute and chronic brucellosis groups was performed using the limma R package. Genes with nominal P value < 0.05 and |logFC| > 0.585 were selected as exploratory candidate differentially expressed genes for downstream analyses. P values were adjusted for multiple testing using the Benjamini–Hochberg method. Because no genes met the threshold of adjusted P value/FDR < 0.05 together with |logFC| > 0.585, the transcriptomic screening results were interpreted as exploratory rather than confirmatory findings. The exploratory candidate gene set was then subjected to functional enrichment analysis using the clusterProfiler R package, including Gene Ontology terms covering biological process, cellular component, and molecular function, as well as Kyoto Encyclopedia of Genes and Genomes pathways.1.5 Weighted gene co-expression network analysis.

Weighted gene co-expression network analysis (WGCNA) was performed to explore co-expression modules associated with clinical phenotypes. After retaining genes with relatively high variability, a gene co-expression network was constructed using the WGCNA R package. Sample clustering was performed to assess potential outliers, and all samples were retained for subsequent analysis. A soft-thresholding power was selected to approximate scale-free network topology. The adjacency matrix was transformed into a topological overlap matrix, and genes were hierarchically clustered based on topological overlap dissimilarity. Gene modules were identified using dynamic tree cutting, and module eigengenes were calculated to summarize the expression profiles of each module. Correlations between module eigengenes and clinical traits were assessed using Pearson correlation analysis. Modules showing relatively strong correlations with acute or chronic brucellosis status were considered phenotype-associated co-expression modules for exploratory downstream analyses.

### 2.5 Feature selection and model construction

**Figure 1:**
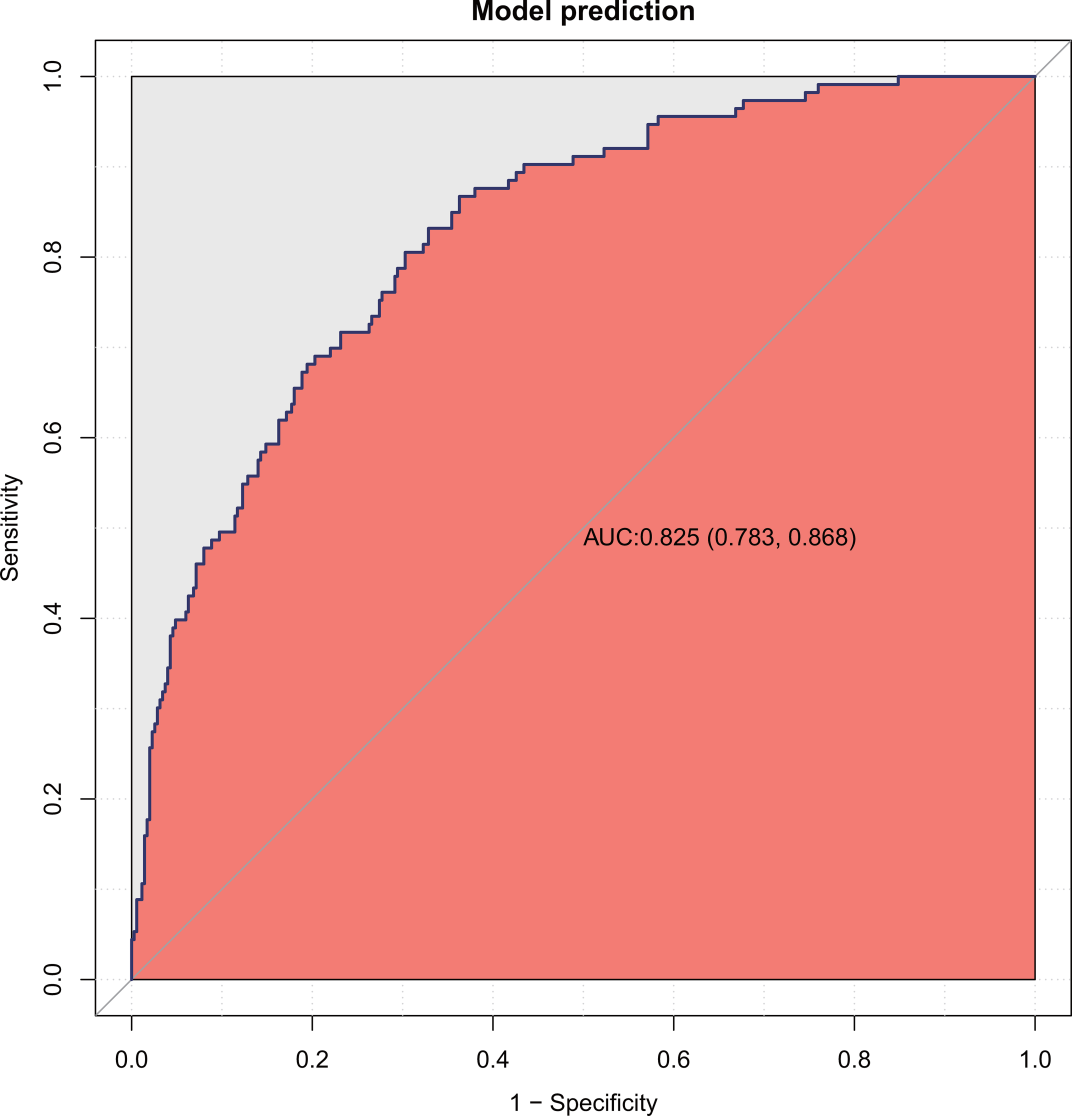
Receiver operating characteristic (ROC) curve of the multivariate logistic regression model predicting chronicity in brucellosis.

Lipid-related genes were obtained from the Reactome ‘Metabolism of lipids’ gene set (REACTOME_METABOLISM_OF_LIPIDS.v2023.2.Hs.gmt). All unique genes included in this curated gene set were defined as lipid-related genes. The overlap between lipid-related genes and exploratory candidate differentially expressed genes was used to identify lipid metabolism-related candidate genes for downstream feature-selection analyses. These candidate genes were then subjected to feature selection by least absolute shrinkage and selection operator (LASSO) regression (R package “glmnet”) and random forest (RF) analysis (R package “randomForest”). For LASSO regression, the expression matrix of candidate lipid metabolism-related genes was used as the predictor matrix, and disease status was used as the binary outcome. LASSO analysis was performed using a binomial family with alpha = 1. The optimal penalty parameter was selected by 10-fold cross-validation using the minimum cross-validated error criterion. Genes with non-zero coefficients at the optimal lambda value were retained as LASSO-selected genes. For random forest analysis, 1,000 trees were constructed, and variable importance was evaluated using the MeanDecreaseGini index. The top 10 genes ranked by MeanDecreaseGini were selected. Genes selected by both methods were retained as hub genes. The final three-gene signature consisted of BDH1, CERS6, and DPEP3. A nomogram integrating these genes was developed using the “rms” package in R to provide individualized risk prediction. Model performance was evaluated by receiver operating characteristic (ROC) curves, calibration curves, and decision curve analysis (DCA) (**Figure 1**). The ROC analysis performed in the original RNA-seq cohort was regarded as an apparent internal performance estimate.

To further evaluate the internal performance of the predefined three-gene model and to reduce optimism bias, additional cross-validation analyses were performed. Because the RNA-seq cohort included only 30 PBMC samples, a conventional training/validation split was not performed, as this would have resulted in very small and unstable subsets. Instead, leave-one-out cross-validation and repeated five-fold cross-validation were used to provide more conservative internal estimates of model performance. For leave-one-out cross-validation, one sample was left out as the test sample in each iteration, and the model was trained using the remaining samples. This process was repeated until each sample had been used once as the test sample. For repeated five-fold cross-validation, stratified five-fold cross-validation was repeated 100 times, and the AUC values across repetitions were summarized. These internal validation analyses were performed for the predefined three-gene model composed of BDH1, CERS6, and DPEP3.

### 2.6 RT-qPCR assay for hub gene expression

For the RT-qPCR assay, an additional set of PBMC samples was obtained from 14 patients with acute brucellosis and 10 patients with chronic brucellosis. Reverse transcription quantitative real-time PCR (RT-qPCR) was performed to further examine the mRNA expression patterns of the candidate hub genes. Total RNA was extracted from PBMCs and reverse-transcribed into cDNA according to the manufacturer’s instructions. The mRNA expression levels of BDH1, CERS6, and DPEP3 were measured by RT-qPCR, with GAPDH used as the reference gene. Each sample was analyzed in technical triplicate. Relative expression levels were calculated using the 2^-ΔΔCt method, with the acute group used as the calibrator group. Statistical comparisons between the acute and chronic groups were performed using ΔCt values.1.8 Immune infiltration, molecular subtyping, and drug–gene interaction analysis.

Immune cell infiltration was estimated using ssGSEA implemented in the GSVA R package, based on predefined immune cell marker sets. Differences between acute and chronic groups were assessed by the Wilcoxon rank-sum test, and correlations between hub genes and immune subsets were examined using Spearman’s method. Molecular subtypes were identified through consensus clustering with the ConsensusClusterPlus R package using hub gene expression profiles, and subtype robustness was validated by principal component analysis (PCA). Differential expression across subtypes was compared by Wilcoxon test. To explore therapeutic potential, hub genes were queried against the DGIdb database (https://dgidb.org/) to retrieve known or predicted drug–gene interactions.

### 2.7 Statistical analysis

Clinical data of 463 patients were analyzed using SPSS 26.0 (IBM, USA) and R software (v4.5.1). Continuous variables were expressed as mean ± standard deviation or median (interquartile range), and compared using Student’s *t* test (parametric) or Mann–Whitney *U* test (non-parametric). Categorical variables were expressed as frequency (%) and compared with the χ² test or Fisher’s exact test. Logistic regression analysis was performed using the “glm” function in R to identify independent predictors.

For transcriptomic analysis, differential expression was assessed using the R package DESeq2, enrichment analysis by clusterProfiler, and feature selection by glmnet (LASSO) and randomForest. Construction of the nomogram and calibration curves was conducted using the R package rms, while predictive performance was evaluated by ROC analysis (“pROC” package) and decision curve analysis (“rmda” package). A two-sided *P* < 0.05 was considered statistically significant.

## 3 Results

Among 463 patients with brucellosis (350 acute and 113 chronic cases), no statistically significant differences were observed between the two groups with respect to sex or age. Fever and hepatic dysfunction were predominant features in the acute group, whereas joint pain, arthritis, and splenomegaly were more frequently observed in the chronic group. In terms of hematological parameters, platelet counts and eosinophil levels were relatively higher in chronic cases. Laboratory testing revealed that patients in the acute group had higher triglyceride (TG) levels, whereas those in the chronic group exhibited elevated high-density lipoprotein cholesterol (HDL-C). In addition, inflammatory markers, including D-dimer, procalcitonin (PCT), and erythrocyte sedimentation rate (ESR), were generally higher in the acute group compared with the chronic group. Overall, the two groups differed in terms of clinical manifestations, inflammatory status, and lipid metabolism (**Table 1**).

**Table 1:** Comparison of baseline characteristics between patients with acute and chronic brucellosis.

Variables	Total (n = 463)	Acute group (n = 350)	Chronic group (n = 113)	*P*
Gender				0.319
Female	136 (29.4)	107 (30.6)	29 (25.7)	
Male	327 (70.6)	243 (69.4)	84 (74.3)	
Age	51.7 ± 14.1	51.3 ± 14.9	53.0 ± 11.1	0.281
Fever	368 (79.5)	302 (86.3)	66 (58.4)	< 0.001
Myalgia	213 (46.0)	152 (43.4)	61 (54)	0.050
Fatigue	318 (68.7)	242 (69.1)	76 (67.3)	0.707
Anorexia	293 (63.3)	228 (65.1)	65 (57.5)	0.144
Headache	79 (17.1)	71 (20.3)	8 (7.1)	0.001
Hepatomegaly	51 (11.0)	42 (12)	9 (8)	0.234
Splenomegaly	214 (46.2)	176 (50.3)	38 (33.6)	0.002
Arthralgia	234 (50.5)	147 (42)	87 (77)	< 0.001
Arthritis	145 (31.3)	90 (25.7)	55 (48.7)	< 0.001
Neurobrucellosis	20 (4.3)	16 (4.6)	4 (3.5)	0.793
Cardiac involvement in brucellosis	6 (1.3)	4 (1.1)	2 (1.8)	0.637
Genitourinary involvement in brucellosis	28 (6.0)	20 (5.7)	8 (7.1)	0.597
WBC, 109/L	4.9 (3.7, 6.6)	4.8 (3.6, 6.5)	5.0 (4.1, 6.6)	0.241
RBC, 1012/L	4.2 (3.8, 4.5)	4.1 (3.8, 4.5)	4.3 (3.9, 4.6)	0.024
HGB, g/L	124.0 (112.0, 136.0)	123.0 (111.0, 134.0)	127.0 (114.0, 144.0)	0.045
PLT, 109/L	206.0 (143.0, 264.0)	196.5 (139.2, 256.0)	222.0 (166.0, 279.0)	0.055
Lymphocytes, 109/L	1.6 (1.2, 2.1)	1.6 (1.2, 2.1)	1.6 (1.4, 2.2)	0.279
Monocytes, 109/L	0.4 (0.3, 0.6)	0.4 (0.3, 0.6)	0.4 (0.3, 0.6)	0.787
Neutrophils, 109/L	2.6 (1.7, 3.8)	2.6 (1.7, 3.8)	2.8 (2.0, 3.7)	0.351
Eosinophil, 109/L	0.0 (0.0, 0.1)	0.0 (0.0, 0.1)	0.1 (0.0, 0.2)	< 0.001
Basophil, 109/L	0.0 (0.0, 0.0)	0.0 (0.0, 0.0)	0.0 (0.0, 0.0)	0.460
ALT, U/L	35.0 (20.0, 61.0)	40.5 (22.2, 70.0)	23.0 (15.0, 39.0)	< 0.001
AST, U/L	32.0 (21.0, 53.0)	36.0 (24.0, 60.8)	24.0 (18.0, 34.0)	< 0.001
TP, g/L	63.4 (58.8, 67.4)	63.5 (59.0, 67.6)	63.3 (58.2, 67.2)	0.748
ALP, U/L	95.0 (73.0, 129.5)	97.0 (73.2, 133.0)	89.0 (71.0, 112.0)	0.079
GGT, U/L	49.0 (26.0, 104.0)	53.0 (29.0, 111.8)	33.0 (21.0, 84.0)	< 0.001
TC, mmol/L	3.7 (3.2, 4.4)	3.7 (3.2, 4.3)	3.8 (3.2, 4.8)	0.115
TG, mmol/L	1.4 (0.9, 1.9)	1.4 (1.0, 1.9)	1.1 (0.8, 1.5)	< 0.001
HDL-C, mmol/L	0.8 (0.7, 1.0)	0.8 (0.7, 1.0)	1.0 (0.8, 1.2)	< 0.001
LDL-C, mmol/L	2.2 (1.8, 2.6)	2.2 (1.8, 2.6)	2.2 (1.8, 2.7)	0.701
PT, s	13.0 (12.1, 13.8)	13.0 (12.2, 13.8)	12.8 (11.9, 13.6)	0.040
D-dimer, mg/L	1.4 (0.6, 5.3)	1.6 (0.7, 6.3)	1.0 (0.4, 3.1)	< 0.001
ESR, mm/h	30.0 (13.0, 50.0)	30.0 (15.0, 50.0)	20.0 (8.0, 49.0)	0.025
PCT, ng/mL	0.1 (0.1, 0.3)	0.2 (0.1, 0.3)	0.1 (0.0, 0.2)	< 0.001
CCP, RU/ml	13.0 (10.3, 16.4)	13.5 (10.3, 17.1)	12.3 (10.3, 14.5)	0.009

The multivariate logistic regression model included 11 variables: fever, joint pain, cardiac involvement, white blood cell count, lymphocyte count, monocyte count, eosinophil count, alanine aminotransferase (ALT), γ-glutamyl transferase (GGT), triglycerides (TG), and high-density lipoprotein cholesterol (HDL-C). The complete multivariable logistic regression results, including regression coefficients, odds ratios, 95% confidence intervals, and P values, are provided in **Supplementary Table 1**. The ROC curve of the model demonstrated an area under the curve (AUC) of 0.825 (95% CI: 0.783–0.868).

**Figure 2:**
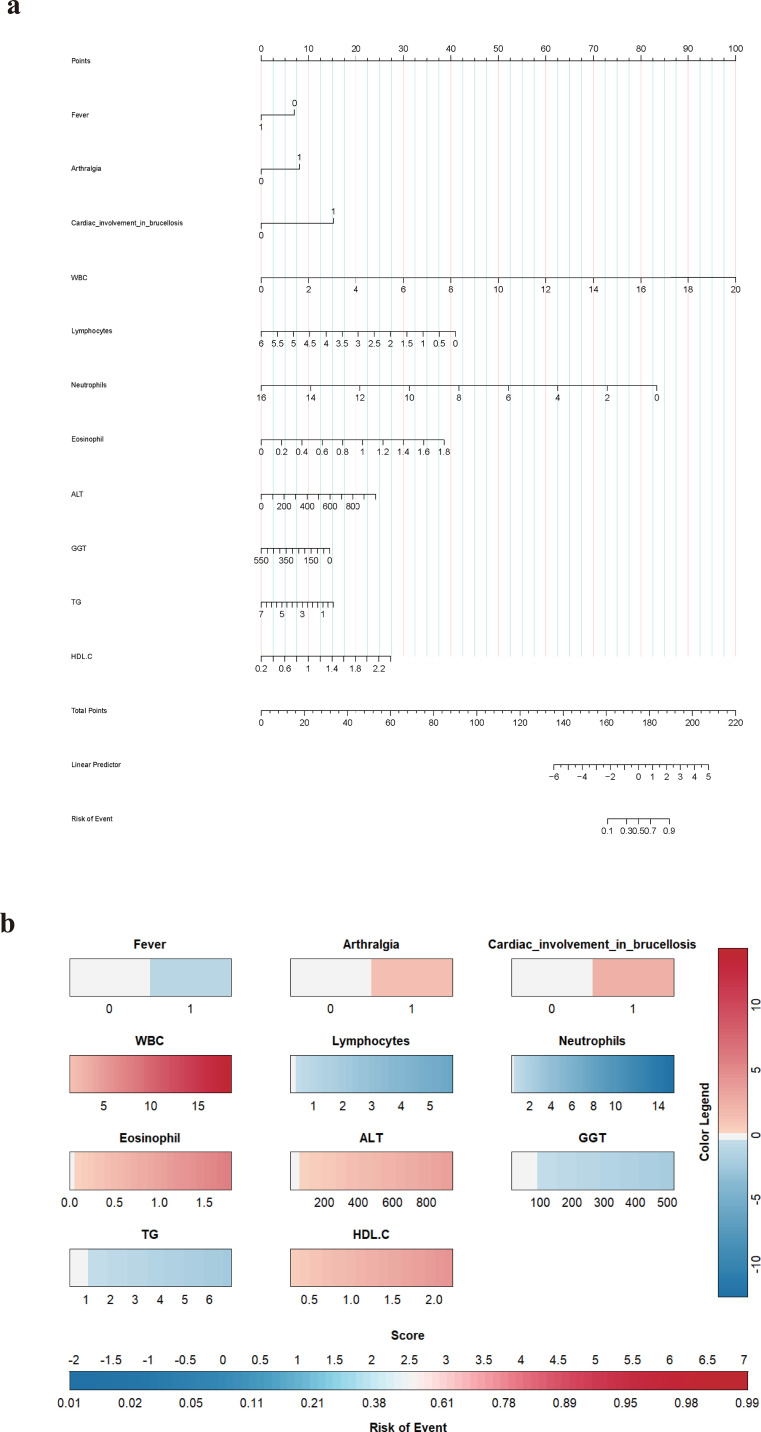
Clinical nomogram predicting the risk of chronicity in brucellosis. **(a)** Nomogram for predicting the risk of chronic brucellosis. **(b)** Color-scale representation of the variables included in the nomogram and the corresponding risk score. (The nomogram was constructed using a multivariable logistic regression model based on selected clinical variables. Continuous variables were scaled as shown, and categorical variables were coded as 0/1. Risk scores represent the predicted probability of chronic brucellosis. Statistical comparisons were performed using χ^2^ test or t-test as appropriate. Confidence intervals (95%) are shown where applicable. The color scales indicate risk levels or measurement values as indicated in the figure.

A nomogram was developed based on the multivariate logistic regression model to provide a visual representation of the risk of chronicity in brucellosis (**Figure 2**). Each variable in the nomogram corresponds to a scoring scale, where values can be selected according to clinical characteristics and converted into individual scores. The sum of these individual scores yields a total score, which can then be mapped to the probability scale at the bottom to estimate the patient’s risk of developing chronicity. The nomogram visually illustrates the relative contribution of each variable within the overall predictive model and may provide a reference for individualized clinical assessment.

### 3.1 Functional characterization of exploratory candidate differentially expressed genes

**Figure 3:**
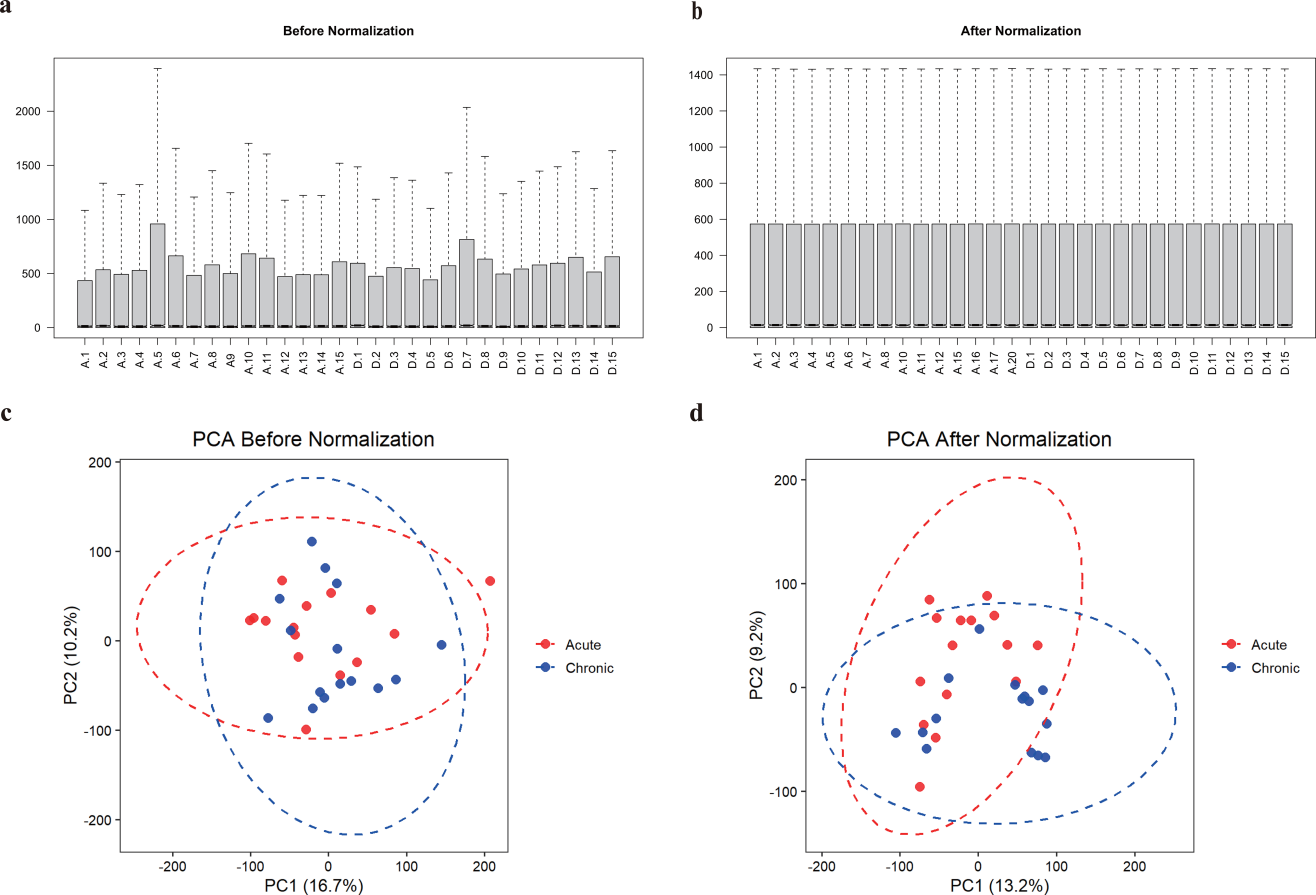
Quality-control assessment of RNA-seq sample distribution **(a)** Expression distribution of samples before normalization. **(b)** Expression distribution of samples after normalization. **(c)** PCA plot of acute (red) and chronic (blue) samples before normalization. **(d)** PCA plot of acute (red) and chronic (blue) samples after normalization.

**Figure 4:**
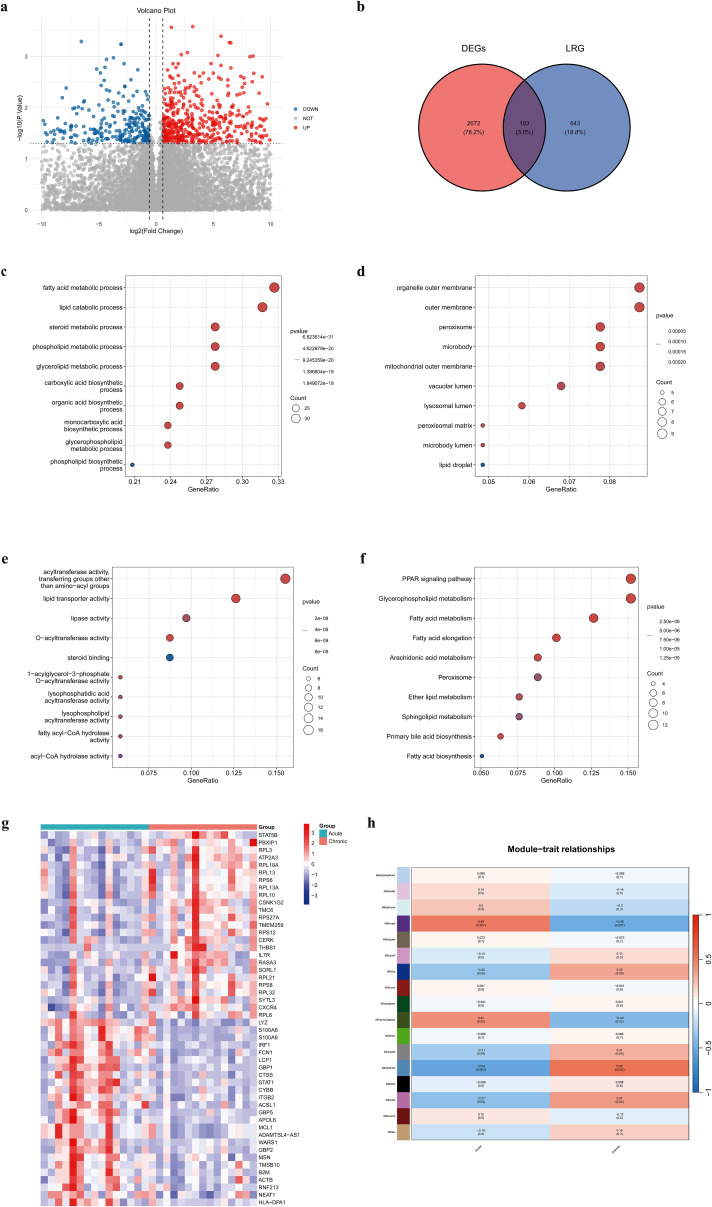
Analyses and identification of exploratory candidate differentially expressed genes between acute and chronic brucellosis patients. **(a)** Volcano plot showing exploratory candidate genes screened using nominal P value < 0.05 and |logFC| > 0.585. P values were adjusted using the Benjamini–Hochberg method; however, no genes reached adjusted P value/FDR < 0.05. **(b)** Venn diagram showing the overlap between exploratory candidate genes and lipid-related genes (LRGs). **(c)** GO enrichment analysis of exploratory candidate genes in the biological process (BP) category. **(d)** GO enrichment analysis of exploratory candidate genes in the cellular component (CC) category. **(e)** GO enrichment analysis of exploratory candidate genes in the molecular function (MF) category. **(f)** KEGG pathway enrichment analysis of exploratory candidate genes. **(g)** Heatmap showing the expression patterns of representative exploratory candidate genes across acute and chronic groups. **(h)** Module–trait relationships between gene co-expression modules and clinical phenotypes.

We analyzed RNA-seq data from 15 acute and 15 chronic brucellosis patients. **Figure 3** was used to visualize sample-level expression distributions and PCA patterns for quality-control assessment before downstream analyses.” To explore transcriptomic differences between the two groups, we performed exploratory differential expression screening. Using nominal P value < 0.05 and |logFC| > 0.585 as exploratory screening thresholds, we identified 2,775 candidate differentially expressed genes, including 1,594 upregulated and 1,181 downregulated genes (**Figure 4**). After Benjamini–Hochberg correction, no genes remained significant at adjusted P value/FDR < 0.05; therefore, these genes were interpreted as exploratory candidates for downstream functional and feature-selection analyses. A Venn diagram demonstrated that 103 genes overlapped between the exploratory candidate gene set and lipid-related genes (LRGs), suggesting a potential association with lipid metabolism (**Figure 4**). GO enrichment analysis showed that these candidate genes were enriched in lipid-related biological processes, such as fatty acid metabolic process, lipid catabolic process, and glycerophospholipid metabolic process (**Figure 4**). In terms of cellular components, these genes were mainly located in peroxisome, lipid droplet, lysosomal lumen, and mitochondrial outer membrane (**Figure 4**). Regarding molecular function, enrichment was observed in acyl-CoA hydrolase activity, lipid transporter activity, and steroid binding (**Figure 4**). KEGG pathway enrichment further highlighted pathways associated with lipid metabolism and immune regulation, including the PPAR signaling pathway, fatty acid metabolism, arachidonic acid metabolism, and glycerophospholipid metabolism (**Figure 4**). Moreover, the heatmap of representative candidate genes displayed distinct expression profiles between acute and chronic groups, reflecting potential transcriptomic differences across clinical phenotypes (**Figure 4**). Finally, the module–trait relationship analysis revealed correlations between specific gene co-expression modules and clinical phenotypes, suggesting transcriptomic modules potentially involved in disease progression (**Figure 4**).2.2 Identification of potential diagnostic genes based on machine learning algorithms and validation of diagnostic hub biomarkers.

**Figure 5:**
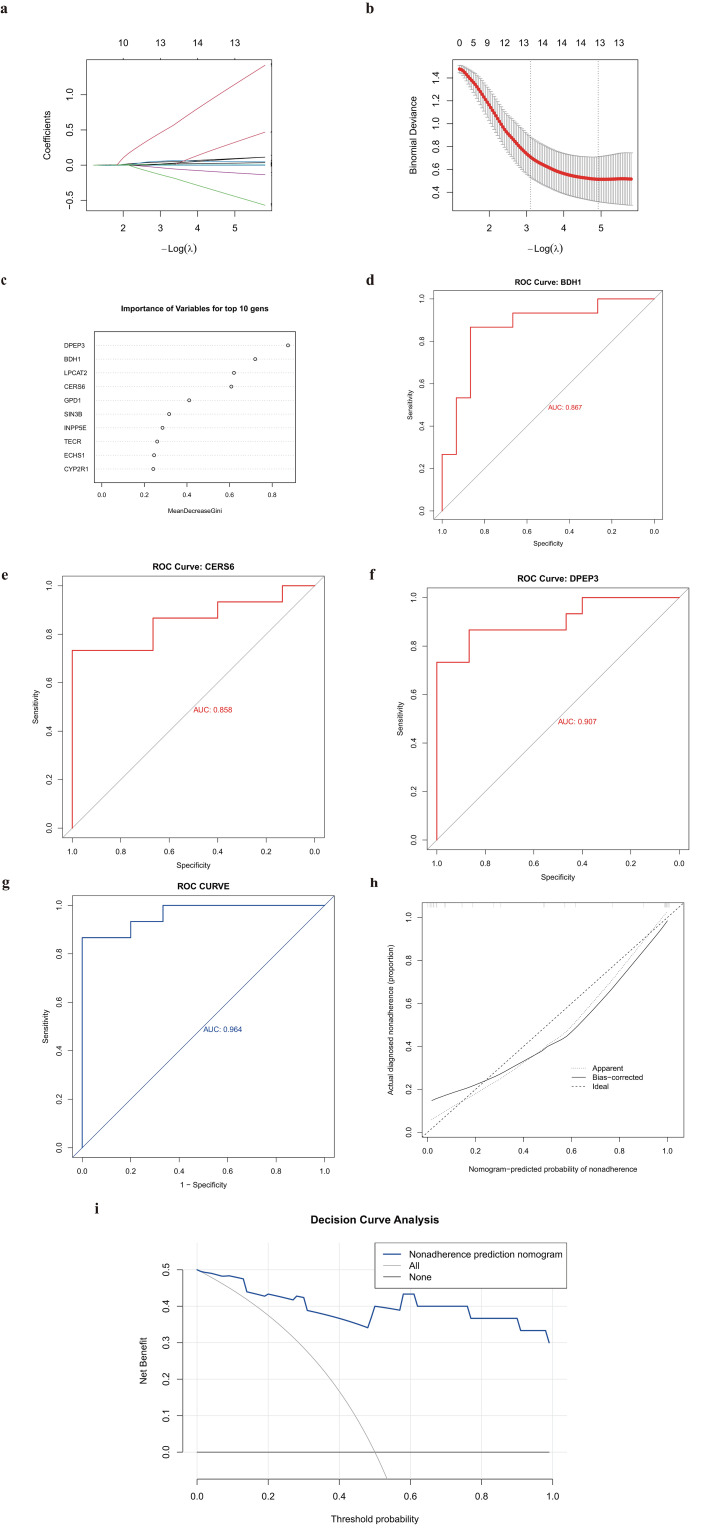
Functional and predictive modeling of lipid metabolism–related DEGs. **(a, b)** Selection of candidate genes using least absolute shrinkage and selection operator (LASSO) regression. **(c)** Random forest analysis ranking the importance of the top 10 candidate genes. **(d)** Receiver operating characteristic (ROC) curve evaluating the predictive performance of BDH1. **(e)** ROC curve evaluating the apparent discriminatory performance of CERS6. **(f)** ROC curve evaluating the apparent discriminatory performance of DPEP3. **(g)** ROC curve evaluating the apparent discriminatory performance of the combined model. **(h)** Calibration plot of the apparent discriminatory nomogram. **(i)** Decision curve analysis (DCA) assessing the clinical net benefit of the predictive model. DEGs, differentially expressed genes; ROC, receiver operating characteristic; DCA, decision curve analysis.

To identify lipid metabolism–related candidate genes with potential discriminatory ability between acute and chronic brucellosis, we applied LASSO regression and random forest analysis. LASSO regression selected three genes with non-zero coefficients (**Figure 5a**), and random forest analysis further highlighted the importance of **BDH1, CERS6,** and **DPEP3** (**Figure 5**).

To preliminarily evaluate the discriminatory performance, ROC curves were generated for each single-gene model and for a combined model; all three single-gene models showed varying degrees of discriminatory ability, and the combined model showed the highest apparent AUC in the internal RNA-seq cohort (**Figure 5d**). Calibration analysis indicated good agreement between predicted and observed outcomes, supporting the robustness of the nomogram (**Figure 5**). Decision curve analysis (DCA) demonstrated greater clinical net benefit for the combined model across a broad range of threshold probabilities compared with single-gene models (**Figure 5**).

### 3.2 Internal evaluation of the three-gene signature

**Figure 6:**
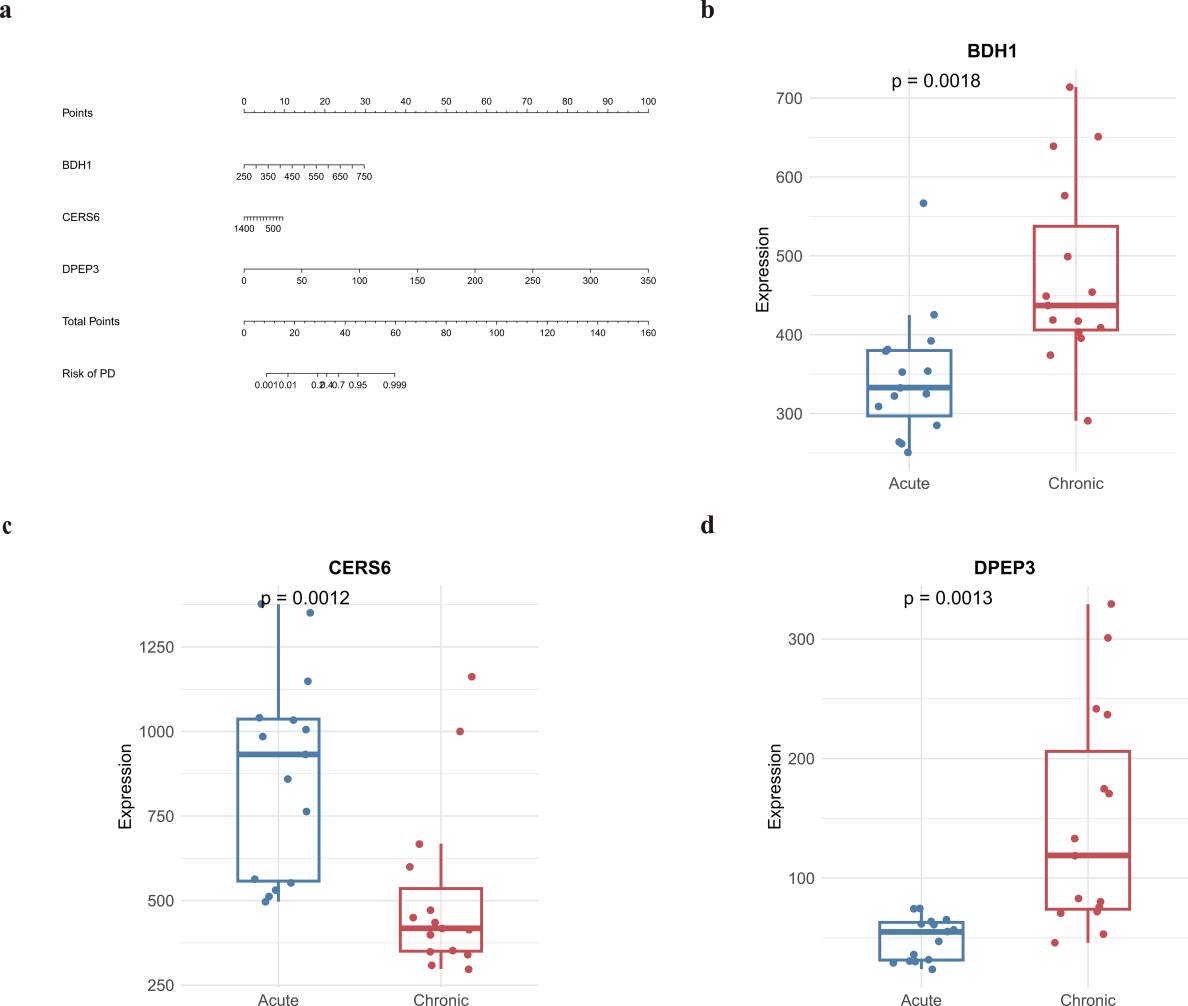
Expression patterns and nomogram construction of the three-gene signature. **(a)** Molecular nomogram for predicting chronic brucellosis based on BDH1, CERS6, and DPEP3 expression. **(b)** Expression level of BDH1 in acute and chronic brucellosis patients. **(c)** Expression level of CERS6 in acute and chronic brucellosis patients. **(d)** Expression level of DPEP3 in acute and chronic brucellosis patients.

To further evaluate the diagnostic value of lipid metabolism–related genes, we constructed a nomogram model incorporating BDH1, CERS6, and DPEP3 expression (**Figure 6**). Expression analysis showed that all three genes differed significantly between acute and chronic brucellosis groups (**Figure 6b**). In the internal RNA-seq cohort, the three-gene model showed a high apparent discriminatory performance, with an AUC of 0.964 (**Figure 5**). Because this apparent AUC was obtained from the same RNA-seq cohort used for model construction, we further performed internal cross-validation to provide more conservative estimates of model performance. Leave-one-out cross-validation yielded an AUC of 0.871 (**Supplementary Figure S1**), and repeated five-fold cross-validation showed a mean AUC of 0.859 ± 0.041, with a median AUC of 0.871 and an interquartile range of 0.831–0.890 (**Supplementary Figure S1**). These results suggest that although the apparent AUC may be optimistic, BDH1, CERS6, and DPEP3 retained exploratory discriminatory potential as a three-gene signature. However, given the limited RNA-seq sample size and the absence of an independent external validation cohort, this signature should be interpreted as exploratory and requires further validation. These results have been added to the Results section and **Supplementary Figure S1**.

### 3.3 RT-qPCR assay for hub gene expression

To further assess the expression patterns of the three candidate hub genes, RT-qPCR was performed using an additional set of PBMC samples from 14 patients with acute brucellosis and 10 patients with chronic brucellosis. After normalization to GAPDH, the relative mRNA expression levels of BDH1, CERS6, and DPEP3 were calculated using the 2^-ΔΔCt method.

**Figure 7:**
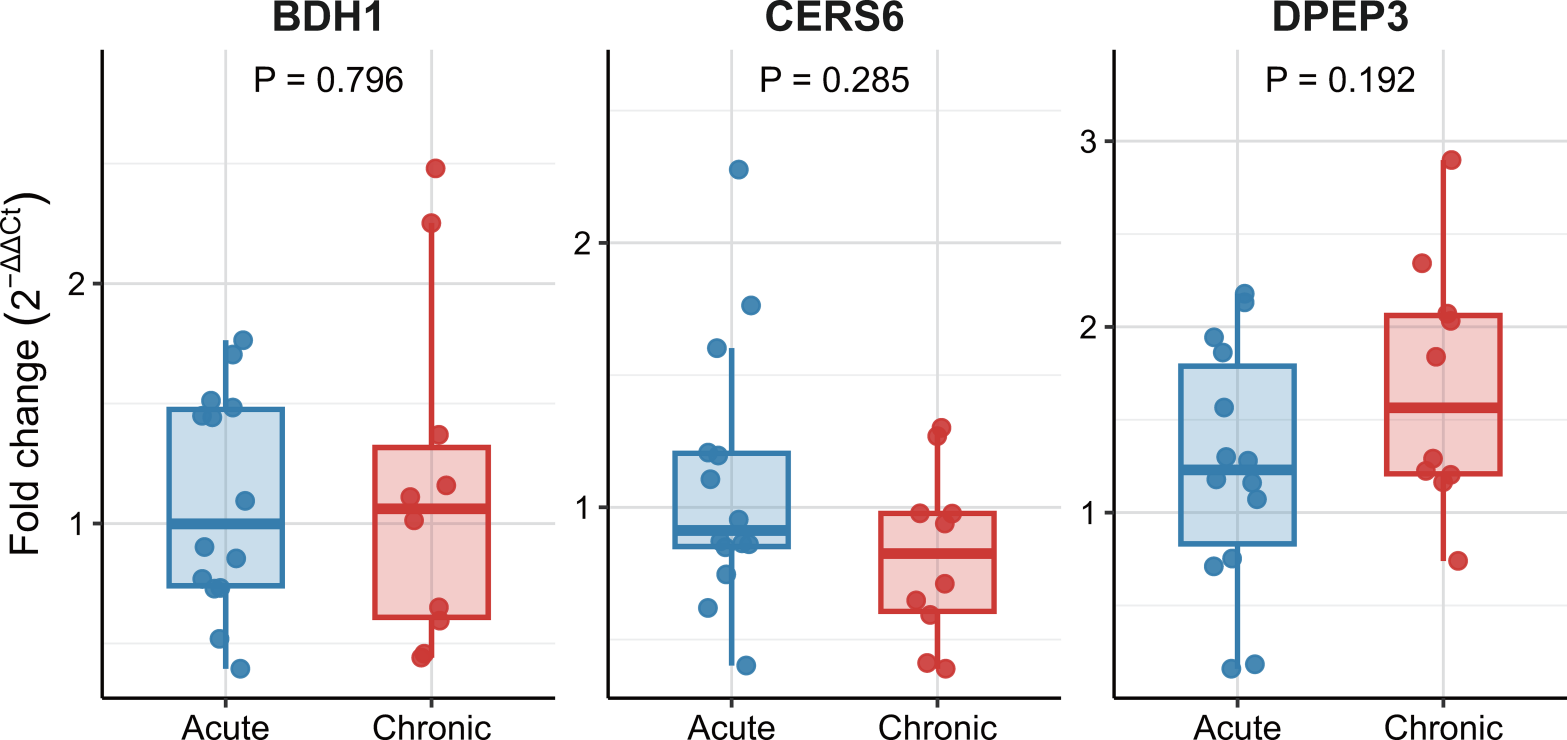
RT-qPCR assay of candidate hub gene expression in PBMCs. The relative mRNA expression levels of BDH1, CERS6, and DPEP3 were measured in PBMC samples from acute and chronic brucellosis patients. Relative expression levels were calculated using the 2^-ΔΔCt method, with GAPDH as the reference gene.

The RT-qPCR results showed expression trends generally consistent with the RNA-seq findings. BDH1 and DPEP3 tended to show higher expression in the chronic group, whereas CERS6 tended to show lower expression in the chronic group. Although the differences did not reach statistical significance, the directionally consistent trends provided preliminary support for the transcriptome-based identification of these candidate hub genes (**Figure 7**).

### 3.4 Group stratification and immune cell infiltration associated with hub genes

**Figure 8:**
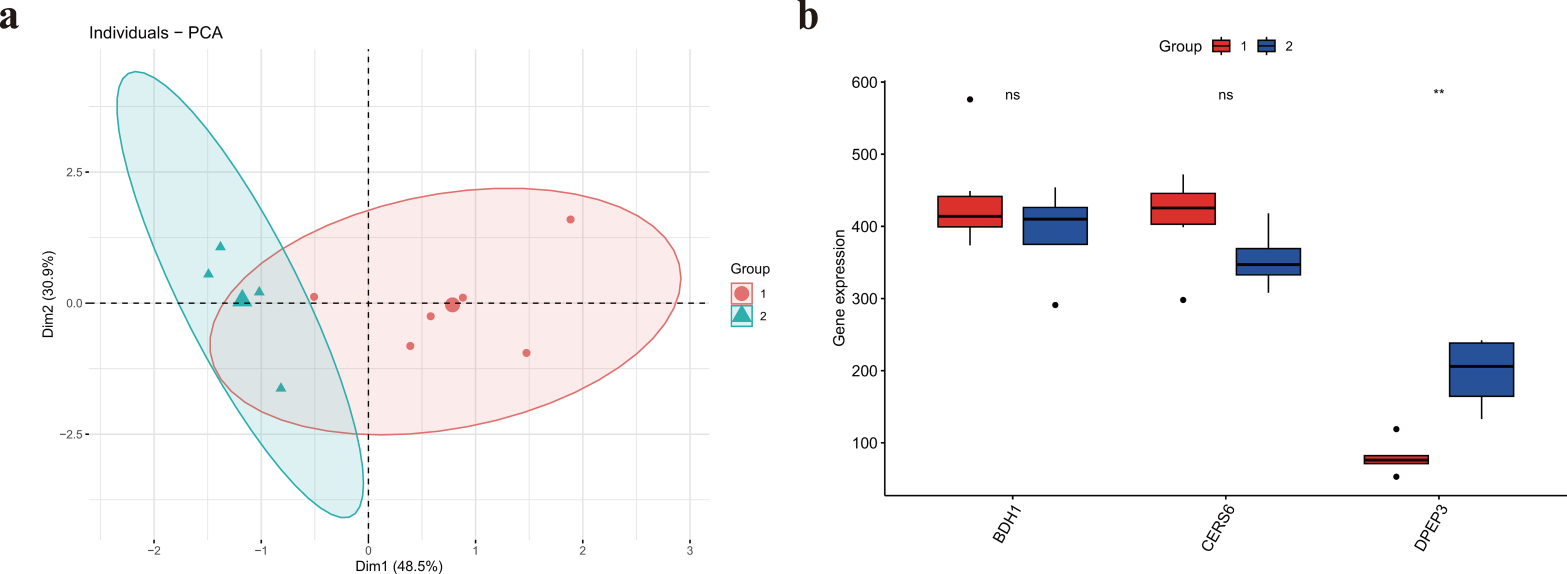
PCA and candidate hub gene expression across molecular subgroups. **(a)** PCA showing the separation of two molecular subgroups derived from consensus clustering based on the expression profiles of BDH1, CERS6, and DPEP3. Group 1 and Group 2 represent the two consensus-clustering-derived molecular subgroups. **(b)** Boxplots showing the expression levels of BDH1, CERS6, and DPEP3 between Group 1 and Group 2. (ns, not significant; **, p < 0.01).

Principal component analysis (PCA) revealed a clear separation between two consensus-clustering-derived molecular subgroups, defined as Group 1 and Group 2 (**Figure 8**), and boxplot analysis further showed that the candidate hub genes BDH1, CERS6, and DPEP3 had distinct expression patterns between the two molecular subgroups, with DPEP3 showing the most significant difference (**Figure 8**). These results suggest that the expression profiles of BDH1, CERS6, and DPEP3 may contribute to exploratory molecular stratification of brucellosis patients.

**Figure 9:**
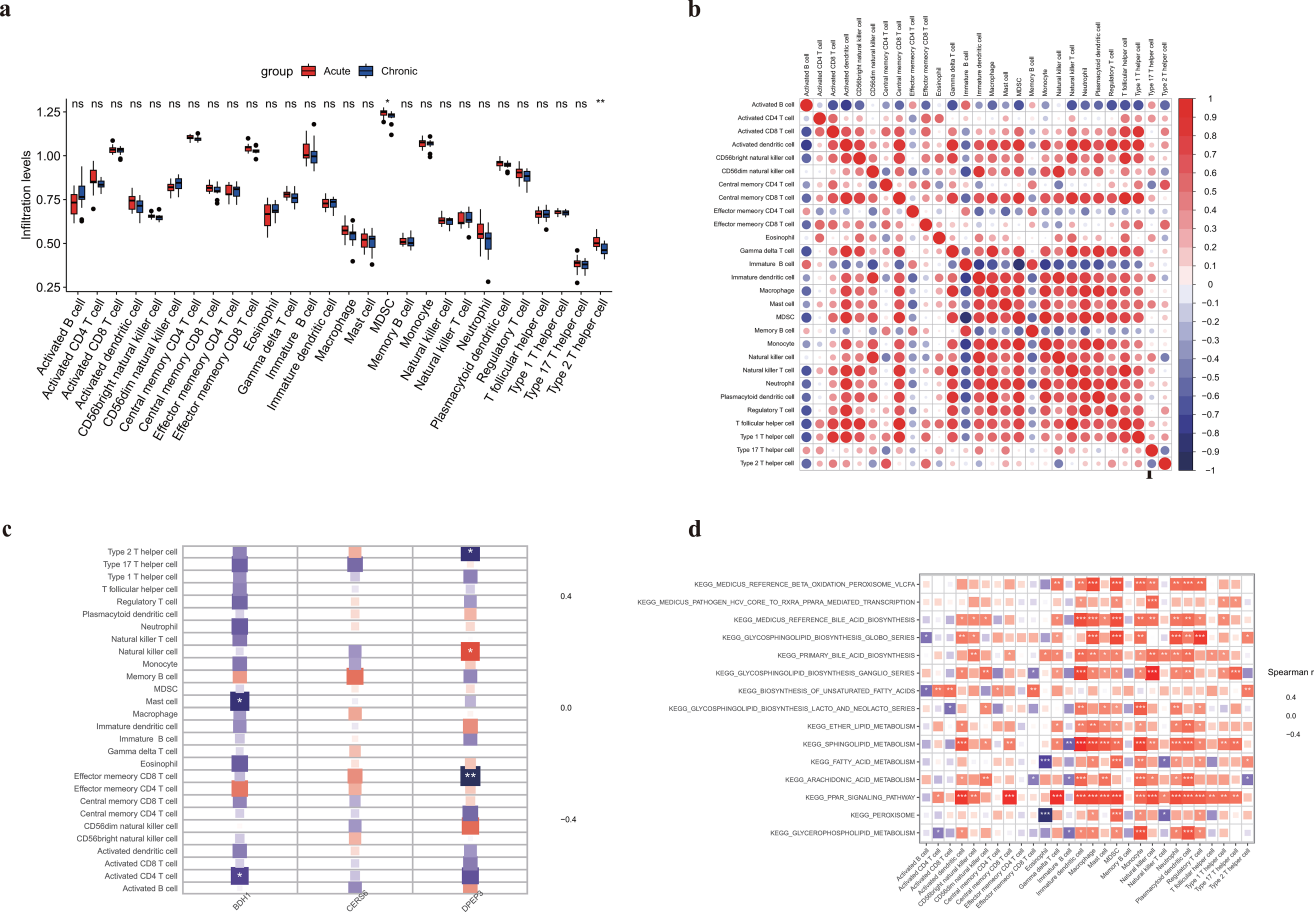
Immune cell infiltration and hub gene associations. **(a)** Differential immune-cell infiltration between acute and chronic groups. **(b)** Correlations among immune-cell subsets. **(c)** Correlations between hub genes (BDH1, CERS6, DPEP3) and immune-cell infiltration **(d)** Correlations between lipid metabolism-related pathways and immune-cell infiltration.

Immune infiltration analysis was then performed to further characterize estimated immune-cell composition between acute and chronic brucellosis groups. The ssGSEA-based analysis showed that most immune-cell subsets did not differ significantly between the two groups, whereas myeloid-derived suppressor cells (MDSCs) and Type 2 T helper cells showed significant between-group differences (**Figure 9**). Correlation analysis revealed coordinated patterns among immune-cell subsets (**Figure 9**), and BDH1, CERS6, and DPEP3 were correlated with specific immune-cell infiltration scores(**Figure 9**). To further explore the relationship between lipid metabolism-related transcriptomic alterations and immune-cell changes, Spearman correlation analysis was performed between ssGSEA scores of lipid metabolism-related pathways and estimated immune-cell infiltration scores. Several lipid metabolism-related pathways, including the PPAR signaling pathway, fatty acid metabolism, arachidonic acid metabolism, sphingolipid metabolism, glycerophospholipid metabolism, and peroxisome-related pathways, showed significant correlations with multiple immune-cell subsets (**Figure 9**). These findings suggest a potential association between lipid metabolic pathway activity and altered immune-cell composition in brucellosis.

### 3.5 Drug–gene interaction analysis of hub genes

Drug–gene interaction analysis indicated that BDH1 is mainly linked to natural products or experimental small molecules, with limited translational potential. DGIdb-based exploratory analysis suggested potential associations between CERS6 and several approved anti-inflammatory and immunomodulatory agents, including TNF-α inhibitors. However, these associations were derived from computational drug–gene interaction prediction and should be interpreted as hypothesis-generating only. They do not demonstrate direct pharmacological interactions or therapeutic efficacy in brucellosis, and further experimental validation is required before any therapeutic relevance can be inferred. No approved or candidate drugs were identified for DPEP3 (**Table 2**).

**Table 2:** Drug–gene interaction analysis of hub genes.

Gene	Drug	Regulatory approval	Indication	Interaction score
BDH1	1-CYCLOHEXYL-N-{[1-(4-METHYLPHENYL)-1H-INDOL-3-YL]METHYL}METHANAMINE	Not Approved		10.44076
BDH1	BIOCHANIN A	Not Approved		0.870063
BDH1	XCT790	Not Approved		1.305095
BDH1	DAIDZEIN	Not Approved		2.61019
BDH1	GENISTEIN	Approved		0.180013
CERS6	CERTOLIZUMAB PEGOL	Approved	DMARD, anti-inflammatory agent	3.728843
CERS6	INFLIXIMAB	Approved	DMARD, anti-inflammatory agent	0.841997
CERS6	ADALIMUMAB	Approved	DMARD, anti-inflammatory agent	0.790967
CERS6	ETANERCEPT	Approved	DMARD, anti-inflammatory agent	1.003919

## 4 Discussion

The differences in lipid metabolism between patients with acute and chronic brucellosis may arise from multilayered mechanistic regulation. In acute infection, inflammatory responses are often accompanied by clinical phenotypes such as reduced high-density lipoprotein cholesterol (HDL-C) and elevated triglycerides (TG), whereas chronic infection may drive long-term dysregulation of the lipid profile (Feingold and Grunfeld, 2000). At the molecular level, immune cells (e.g., PBMCs) undergo lipid metabolism-related alterations during different stages of infection—for instance, type I interferon (IFN-I) signaling in the acute phase regulates the expression of lipid metabolism–related genes and the production of antiviral lipids, whereas in the chronic phase, the reprogramming shifts toward energy maintenance and immunosuppressive mechanisms (Palmer, 2022). Similarly, in models of acute exacerbation of chronic obstructive pulmonary disease (COPD), lipid metabolic signatures have been shown to vary according to disease status (Wang et al., 2023). In viral infections, patients with COVID-19 exhibit dyslipidemia—such as decreased HDL-C and increased TG—that correlates significantly with disease severity (Kowalska et al., 2022). These findings provide important insights for further elucidating the mechanistic differences in lipid metabolic states between acute and chronic brucellosis.

Increasing evidence from empirical studies indicates that the peroxisome proliferator-activated receptor (PPAR) pathway plays a crucial role in anti-inflammatory regulation, immune modulation, and metabolic homeostasis during chronic infections. Díaz et al. reported that PPARγ expression was elevated in PBMCs from tuberculosis patients and further enhanced in macrophages upon *Mycobacterium tuberculosis* stimulation. Activation of PPARγ suppressed cytokines such as IL-1β and IL-10, suggesting that PPARγ exerts critical immunomodulatory effects in chronic infection (Díaz et al., 2023). Moreover, Yu et al. demonstrated that the STAT6/IL-4/IL-13 axis induces PPARγ expression, which in turn promotes M2 polarization and anti-inflammatory effects through downstream regulation of arginase-1 (Arg1). These findings further underscore the role of PPARγ as a central metabolic–immune crosstalk regulator within infectious immune environments (Yu et al., 2023). Similarly, Fantacuzzi et al. highlighted in studies of viral infections that PPAR agonists exhibit anti-inflammatory, antioxidant, and immunomodulatory properties, and have been proposed as potential adjunctive therapeutic targets in the context of SARS-CoV-2 infection (Fantacuzzi et al., 2022).

Xavier et al. demonstrated that during chronic brucellosis, PPARγ expression is markedly elevated in M2 macrophages, driving host energy metabolic reprogramming. This process enhances glucose uptake and fatty acid oxidation, which may supply a continuous carbon source and energy supply for intracellular bacteria. Pharmacological interventions revealed that PPARγ inhibitors (e.g., GW9662) reduced the expression of M2 markers and decreased bacterial burden, whereas PPARγ agonists (e.g., rosiglitazone) promoted the long-term survival of *Brucella* within host cells. These findings suggest that PPARγ, as a metabolic regulator, plays a pivotal pathogenic role in sustaining chronic *Brucella* infection and may represent a promising target for immunomodulatory interventions (Xavier et al., 2013).

Fatty acid oxidation is increasingly recognized as an important metabolic process involved in immune-cell regulation, with context-dependent roles in promoting either inflammatory or anti-inflammatory immune responses (Kemp et al., 2024). Although direct studies on BDH1, DPEP3, and CERS6 in brucellosis models are currently lacking, existing evidence supports their potential roles within infection-related metabolic–immune networks. BDH1 is a key enzyme in ketone metabolism. Although expressed at very low levels in macrophages, the ketone pathway has been shown to participate in inflammatory regulation and T-cell functional recovery, suggesting that BDH1 may influence energy supply and immune tolerance mechanisms during chronic infection (Goldberg et al., 2023). CERS6 catalyzes the generation of C16 ceramide, serving as a critical node in inflammatory mediation and immune signaling. Elevated CERS6 expression has been reported to enhance the release of nitric oxide (NO) and tumor necrosis factor-α (TNF-α), as well as to modulate tissue injury in intestinal inflammation models (Schiffmann et al., 2012). DPEP3 has not yet been reported in the context of infectious diseases; however, its membrane-anchored structure suggests a potential role in modulating the immune microenvironment, warranting further investigation. Although direct studies on BDH1, DPEP3, and CERS6 in brucellosis models are currently lacking, existing evidence suggests that these genes may be involved in infection-related metabolic–immune networks. Therefore, these genes should be regarded as candidate lipid metabolism-related feature genes requiring further functional validation.

The chronicity of brucellosis may be accompanied by immune cell metabolic dysregulation, highlighting opportunities for metabolism-targeted interventions. Recent evidence indicates that metabolic pathways, including glycolysis, oxidative phosphorylation, and fatty acid oxidation, can influence immune-cell phenotypes and functions, which may connect metabolic remodeling to immune regulation in disease (Hu et al., 2024). Single-cell studies have revealed stage-specific remodeling of immune cell lineages and functions across different phases of brucellosis, supporting the concept of disease course–related immunometabolic reprogramming (Wang et al., 2024b). In the chronic phase, patients exhibit expansion of peripheral myeloid-derived suppressor cells (MDSCs) along with activation of immunosuppressive metabolic axes such as ARG1, suggesting that metabolic immunosuppression contributes to persistent infection (Hou et al., 2024). At the host metabolic level, restricting glucose flux or increasing itaconate has been shown to inhibit *Brucella* growth (Lacey et al., 2021). Meanwhile, upregulation of the lipid-sensing receptor TREM2 supports the maintenance of the M2 phenotype and promotes chronicity; targeting TREM2 reduces bacterial burden and has been proposed as an adjunctive target to antibiotics (Wang et al., 2024). In addition, modulation of lipid peroxidation and ferroptosis pathways can also significantly affect intracellular bacterial survival (Zhang et al., 2024). Collectively, these lines of evidence indicate that targeting immunometabolic nodes—such as TREM2, the itaconate/glucose-restriction pathway, and lipid peroxidation/ferroptosis—represents a feasible and biologically grounded strategy for metabolic intervention in brucellosis.

Recent studies have shown that brucellosis is not only associated with macrophage M1/M2 polarization but also involves multiple immunometabolic mechanisms. In infected individuals, the STING–HIF-1α pathway drives metabolic reprogramming to sustain inflammatory responses (Gomes et al., 2021). *Brucella* exploits lipid rafts to mediate cellular invasion (Wang et al., 2024b); and it modulates host cell fate via ferroptosis pathways to facilitate replication and release (Zhang et al., 2024). Moreover, host lipids such as linoleic acid have been shown to reduce bacterial survival (Zhang et al., 2024), while systemic metabolomic profiling has revealed pronounced alterations in lipid metabolism during the chronic stage of disease (Fu et al., 2024). These studies provide useful background for interpreting the lipid metabolism-related and immunometabolic features observed in chronic brucellosis, but they do not establish causal mechanisms in the present cohort. We further performed an exploratory correlation analysis between lipid metabolism-related pathway ssGSEA scores and immune-cell infiltration scores. The observed correlations suggested that lipid metabolism-related transcriptional alterations may be associated with immune-cell composition. However, because the analysis was based on bulk PBMC transcriptomics, these findings should be interpreted as exploratory associations rather than evidence of direct mechanistic regulation or immune-cell functional changes. This study has several strengths. First, it included a large clinical cohort of more than 400 patients with brucellosis, substantially enhancing the statistical power and W@Areliability of the findings. Second, by integrating clinical lipid profiles with PBMC transcriptomic data, we performed a multilayered analysis that systematically identified lipid metabolism-related features associated with chronic brucellosis, from the clinical to the molecular level.

From a translational perspective, the BDH1–CERS6–DPEP3 signature should currently be regarded as an exploratory candidate biomarker panel rather than a ready-to-use clinical diagnostic tool. Realistic clinical implementation would require further validation in larger independent and multicenter cohorts, development of a standardized and clinically feasible assay such as RT-qPCR, determination of reproducible cut-off values, and prospective assessment of predictive performance. If validated, this three-gene signature may be integrated with clinical variables to assist chronicity risk stratification and follow-up decision-making in patients with brucellosis. However, before such application, external validation and protein-level or functional confirmation remain necessary.

Nonetheless, this study has several limitations. First, this was a single-center study, which may introduce potential selection bias and limit the generalizability of the findings. In addition, longitudinal sampling was not performed; therefore, the dynamic changes in clinical lipid profiles, PBMC transcriptomic features, and immune-cell composition during disease progression or treatment could not be assessed. Second, the RNA-seq cohort was relatively small, including 15 acute and 15 chronic brucellosis cases, and a formal prospective power calculation was not performed because the analysis was exploratory and constrained by the availability of qualified pretreatment PBMC samples. Given the limited sample size, a conventional training/validation split was not performed, as this would have resulted in very small and unstable subsets. The originally reported AUC of 0.964 should therefore be interpreted as an apparent internal performance estimate and may be affected by overfitting and optimism bias. To provide a more conservative assessment, we performed internal validation of the predefined BDH1–CERS6–DPEP3 model using leave-one-out cross-validation and repeated five-fold cross-validation. However, these analyses remain internal validation and cannot replace external validation in an independent cohort. Therefore, the transcriptomic findings and machine learning-derived three-gene signature should be interpreted cautiously and require validation in larger, independent cohorts. In addition, although 2,775 candidate differentially expressed genes were identified using nominal P value < 0.05 and |logFC| > 0.585 as exploratory screening thresholds, no genes remained significant after Benjamini–Hochberg FDR correction, further supporting the need for cautious interpretation of these transcriptomic findings. Because this study relies on bulk PBMC transcriptomics, some observed transcriptomic differences between acute and chronic brucellosis may reflect changes in immune-cell composition rather than intrinsic transcriptional rewiring. Our ssGSEA-based immune-cell estimation provides complementary insights into relative immune-cell abundances, and the correlations between lipid metabolism-related pathway scores and immune-cell infiltration scores should be interpreted as exploratory and potentially influenced by cell composition. Future studies using single-cell transcriptomics or sorted cell populations are needed to disentangle intrinsic transcriptional changes from shifts in cellular composition. Third, external independent cohorts and functional experiments were lacking to confirm the causal roles of the three identified genes in chronic progression. Although we further assessed the mRNA expression levels of BDH1, CERS6, and DPEP3 by RT-qPCR using an additional set of PBMC samples, these results should be interpreted as preliminary supportive evidence rather than definitive validation. The RT-qPCR results showed directionally consistent expression trends with the RNA-seq data; however, the differences did not reach statistical significance, which may be related to the limited sample size, inter-individual heterogeneity of clinical samples, and the complexity of PBMC composition. In addition, RT-qPCR evaluates mRNA expression rather than protein expression, and Western blot analysis could not be performed because of the limited amount of available PBMC material. Therefore, larger independent cohorts, protein-level validation, and functional experiments are still needed to confirm the predictive performance and biological roles of the three-gene model. Fourth, the drug–gene interaction analysis was based on DGIdb and should be regarded as exploratory and hypothesis-generating. The predicted association between CERS6 and several approved anti-inflammatory and immunomodulatory agents, including TNF-α inhibitors, does not demonstrate direct pharmacological interactions or therapeutic efficacy in brucellosis. Further cellular, animal, and clinical studies are required to validate the biological and therapeutic relevance of these predicted drug–gene interactions. Finally, important confounding variables that can influence lipid metabolism, including diet, BMI, pre-existing metabolic disorders, and medication use, were not fully controlled or adjusted in this study. Although all participants were recruited from the same clinical center and standard protocols were applied, future studies should systematically account for these factors to more accurately characterize lipid metabolism alterations associated with chronic brucellosis.

Future studies should further validate the proposed three-gene signature in multicenter, large-scale clinical cohorts to enhance its reliability and generalizability. In addition, multi-omics approaches, such as lipidomic and metabolomics, together with functional experiments, are needed to more precisely characterize the dynamic associations between lipid metabolic alterations and chronic brucellosis progression. Ultimately, these efforts may provide more robust experimental and clinical evidence for understanding metabolism-related host-response patterns in brucellosis.

## 5 Conclusion

This study integrated clinical data and PBMC transcriptomic analysis to explore lipid metabolism-related features associated with chronic brucellosis. In an exploratory RNA-seq cohort, candidate lipid metabolism-related transcriptomic differences were observed between acute and chronic brucellosis, and enrichment analysis highlighted pathways such as PPAR signaling. The BDH1–CERS6–DPEP3 signature may provide a candidate molecular feature for future chronicity risk stratification, but its clinical utility requires validation in larger independent cohorts. Overall, these findings provide hypothesis-generating evidence that lipid metabolism-related transcriptomic alterations and immune-cell composition changes may be associated with chronic brucellosis.

## Data Availability

The data analyzed in this study is subject to the following licenses/restrictions: The datasets generated and analyzed during the current study are not publicly available due to institutional and legal restrictions related to patient privacy and the terms of the ethics approval (Ethics Committee of the First Hospital of Shanxi Medical University, Approval No. KYYJ-2025-143; consent for retrospective chart review was waived). Deidentified data, data dictionaries, and analytic codes will be made available to qualified researchers upon reasonable request by the corresponding author, subject to a data use agreement and approval by the Ethics Committee. Requests should include a brief proposal and will be evaluated within approximately 2-4 weeks. Requests to access these datasets should be directed to Rong Wang wangrong_0920@163.com.

## Supplementary material

Supplementary Table 1 Multivariable logistic regression analysis of clinical variables associated with chronic brucellosis Note: Interaction score was retrieved from DGIdb, which integrates multiple drug–gene interaction resources. “Indication/Drug class” is based on approved clinical use or common pharmacological classification where available, and “experimental small molecule/natural product” is used for compounds without approved indications. These interactions should be interpreted as exploratory and hypothesis-generating, and do not represent experimentally validated pharmacological interactions in brucellosis

Supplementary Figure 1 Internal cross-validation of the three-gene signature. **(a)** ROC curve of the predefined BDH1–CERS6–DPEP3 model using leave-one-out cross-validation (AUC = 0.871). **(b)** AUC distribution from 100 repeats of stratified five-fold cross-validation (mean AUC = 0.859 ± 0.041).
