# Supplementary figures and images for: Integrated clinical and PBMC transcriptomic profiling identifies lipid metabolism-related candidate signatures associated with chronic brucellosis
Source: Front Cell Infect Microbiol. 2026 Jul 2;16:1725128. doi: 10.3389/fcimb.2026.1725128 (PMC13372603; doi:10.3389/fcimb.2026.1725128)

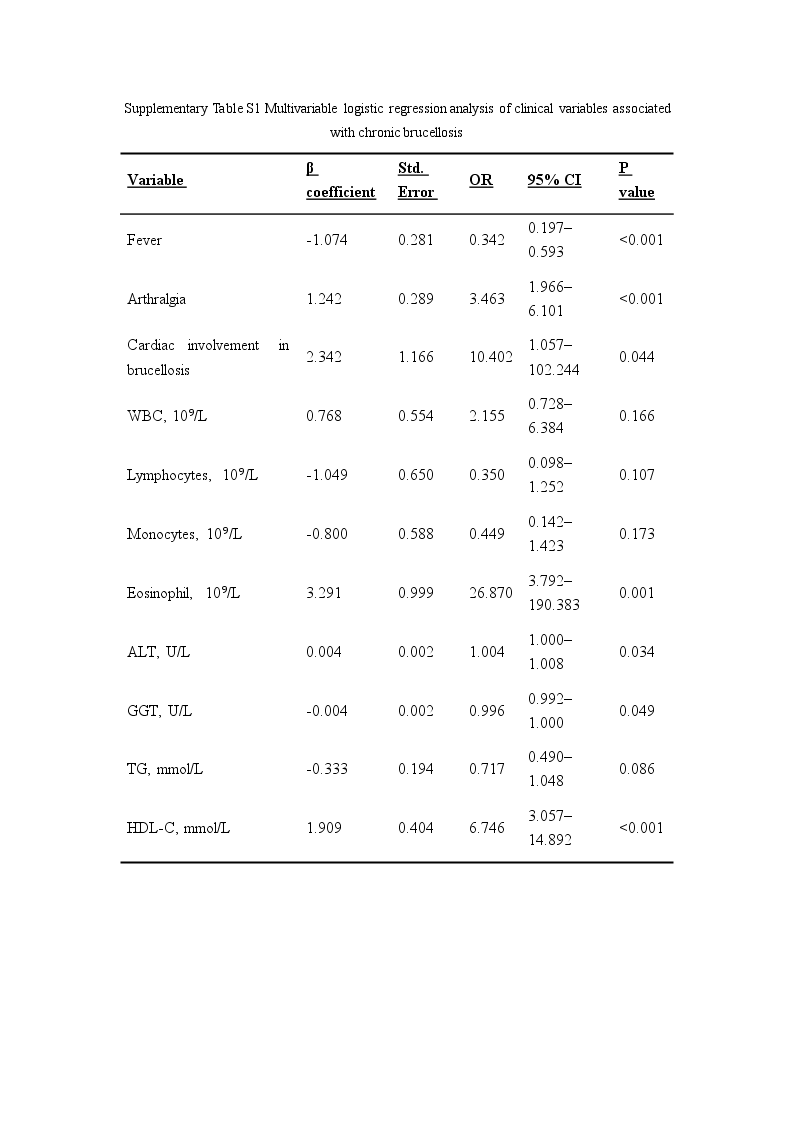

Supplement: Supplementary Table 1 — Multivariable logistic regression analysis of clinical variables associated with chronic brucellosis Note: Interaction score was retrieved from DGIdb, which integrates multiple drug–gene interaction resources. “Indication/Drug class” is based on approved clinical use or common pharmacological classification where available, and “experimental small molecule/natural product” is used for compounds without approved indications. These interactions should be interpreted as exploratory and hypothesis-generating, and do not represent experimentally validated pharmacological interactions in brucellosis [file Image1.tiff]

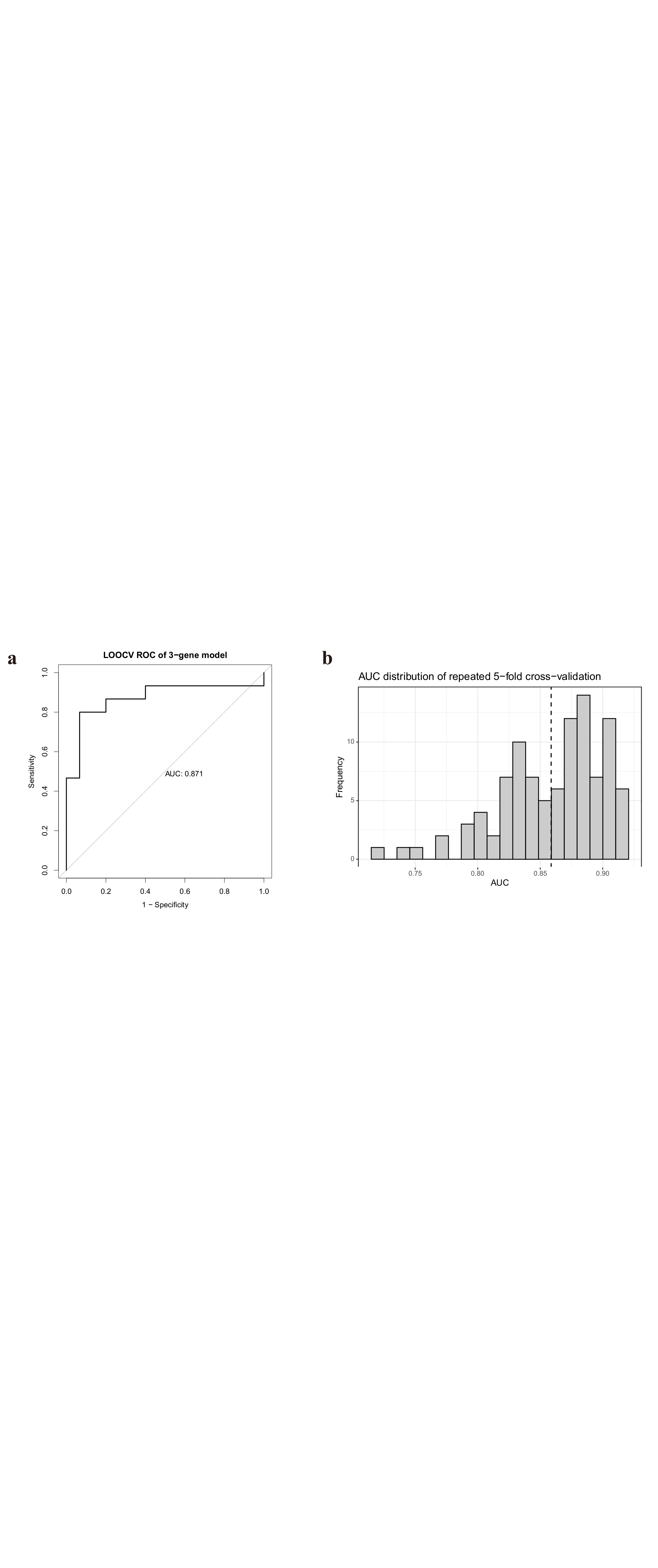

Supplement: Supplementary Figure 1 — Internal cross-validation of the three-gene signature. (a) ROC curve of the predefined BDH1–CERS6–DPEP3 model using leave-one-out cross-validation (AUC = 0.871). (b) AUC distribution from 100 repeats of stratified five-fold cross-validation (mean AUC = 0.859 ± 0.041). [file Image2.tif]
